# Periodontitis, colorectal cancer and associated factors: A cross-sectional study

**DOI:** 10.4317/jced.63576

**Published:** 2026-01-28

**Authors:** Rosita Elena Espejo-Carrera, María Elizabeth Mendoza-Rubio, Carlos Alberto Minchón-Medina, César Abraham Vásquez-Plasencia, José Antonio Caballero-Alvarado, César León-Vega, Oscar Martín Del Castillo-Huertas, Angel Steven Asmat-Abanto

**Affiliations:** 1 Master of Science in Clinical Research. Professor of Stomatology Study Program, Antenor Orrego Private University, Trujillo, Peru; 2 Student of Stomatology Study Program, Antenor Orrego Private University, Trujillo, Peru; 3 Doctor in Public Health. Director of Innovation and Technological Transfer. Professor of the Posgraduate School. Professor of Statistics Study Program. National University of Trujillo, Trujillo, Peru; 4 Master in Stomatology. Specialist in Periodontics. Professor of Stomatology Study Program, Antenor Orrego Private University, Trujillo, Peru; 5 School of Medicine, Antenor Orrego Private University, Trujillo, Peru. Department of Surgery, Regional Docente de Trujillo Hospital, Trujillo, Peru. PhD in Clinical and Translational Research; 6 Professor of Human Medicine Study Program, Antenor Orrego Private University, Trujillo, Peru; 7 Doctor in Stomatology. Specialist in Orthodontics and Maxillary Orthopedics. Director of Stomatology Study Program, Antenor Orrego Private University, Trujillo, Peru; 8 Doctor in Stomatology. Specialist in Periodontics. Professor of Human Medicine Study Program, Antenor Orrego Private University, Trujillo, Peru. Professor of Stomatology Study Program, Antenor Orrego Private University, Trujillo, Peru. Visiting Professor of Posgraduate School, Señor de Sipán University, Chiclayo, Peru

## Abstract

**Background:**

Periodontitis can cause systemic alterations associated with various oncological diseases, including colorectal cancer (CRC), with little information on this possible association and its related factors. Objectives: To determine the association between periodontitis severity, CRC, and related factors in adult patients.

**Material and Methods:**

This cross-sectional study was conducted between January and March 2025 at the Trujillo Regional Teaching Hospital and the "Dr. Luis Pinillos Ganoza" Regional Institute of Neoplastic Diseases - North IREN, Trujillo, Peru. A total of 320 adult patients were evaluated: 160 without colorectal cancer (non-CRC) and 160 with colorectal cancer (CRC). Inter- and intra-rater reliability was determined for the diagnosis of periodontitis, bleeding on probing (BOP), and dental plaque control (DPC). The corresponding specialist physician performed the diagnosis of colorectal cancer (CRC). Statistical analysis was performed using the Chi-square test, ordinal logistic regression, and binary logistic regression models, with a significance level of p &lt; 0.05.

**Results:**

No association was found between the severity of periodontitis and CRC diagnosis in the evaluated population (p=0.068). The most common CRC stages were stage IV (48.1%) and stage III (39.4%), and the most common tumor locations were the rectum (40.0%) and sigmoid colon (36.3%).

**Conclusions:**

There is no association between the severity of periodontitis and CRC diagnosis.

## Introduction

Periodontitis is a chronic inflammatory condition associated with an imbalance in the oral microbiome. This condition causes gradual, permanent deterioration of the tissues that support the teeth, potentially leading to tooth mobility and tooth loss (1). Worldwide, it is estimated that 45%-90% of individuals have some degree of periodontitis, with its severe form affecting 11.2%-20% of the population (2). Nearly one-third of recent studies on periodontitis have focused on exploring its link to various systemic diseases, including diabetes mellitus, cardiovascular and respiratory diseases, Alzheimer's disease, and some types of cancer (3 , 4). Cancer is a disease characterized by the unregulated growth of abnormal cells, which evolve through natural selection via genetic and epigenetic alterations, developing a potentially lethal phenotype (3). Colorectal cancer (CRC) represents one of the leading causes of disease and death worldwide. Globally, it is the second most common malignant neoplasm with the highest number of deaths in both sexes (1), with a strong association with age, being more common in people over 80 years of age, with a less favorable prognosis (5). Several factors have been linked to the development of CRC, such as male gender, low socioeconomic status, presence of diabetes mellitus, excess body fat, family history of CRC in first-degree relatives, smoking habits, high intake of red and processed meats, and excessive alcohol consumption, among others (4). Specifically, two periodontopathogenic bacteria have been identified in CRC: Fusobacterium nucleatum (F. nucleatum) and Porphyromonas gingivalis (P. gingivalis). F. nucleatum adheres to tumor cells and fibroblasts via its surface proteins FadA, Fap2, and RadD, and induces the production of inflammatory factors, suppressing the immune cellular function of macrophages and T and NK lymphocytes, contributing to the development of CRC (4 , 6). In addition, it has been associated with regional lymph node metastasis (1). P. gingivalis can alter the balance of the intestinal microbiota, elevating blood endotoxin concentrations, inducing a systemic inflammatory response, modifying host metabolism, and facilitating evasion of the immune system, thus generating favorable pathophysiological conditions for colorectal carcinogenesis (7). Based on the above, this study aimed to determine the association between periodontitis and CRC in adult patients, while accounting for potentially confounding factors, including sociodemographic characteristics, diverticulosis, type 2 diabetes mellitus (T2DM), cigarette and alcohol consumption, dental plaque control (DPC), and bleeding on probing (BOP). The findings of this research aim to provide relevant information to understand better periodontal medicine and health in relation to oncological diseases. It also motivates future researchers to explore potential clinical links that may promote periodontal health and develop interventions that improve the prognosis and quality of life of cancer patients.

## Material and Methods

This cross-sectional study was conducted at the Department of Surgery of the Trujillo Regional Teaching Hospital (TRTH) and the Department of Abdominal Surgery of the "Dr. Luis Pinillos Ganoza" North Regional Institute of Neoplastic Diseases (North IREN), Trujillo, Peru, between January and March 2025. The sample consisted of 320 patients: 160 with CRC and 160 without CRC (non-CRC). The sample size was calculated using the formula for comparing patients with CRC and non-CRC using data generated through a pilot study conducted with 30 patients per group and with the following parameters: n (Sample size for each group), Z/2=1.96 (Normal value with type I error of =5%), Z=1.645 (Normal value with test power of 1-=95%), p1=0.830 (Prevalence of moderate or severe periodontitis in CRC patients, estimated based on the pilot sample), p1=0.655 (Prevalence of moderate or severe periodontitis in non-CRC patients, estimated based on the pilot sample), p = 0.743 (Prevalence of moderate or severe periodontitis in CRC and non-CRC patients). The selection method was non-probabilistic and accidental. The study included adult patients aged 40 - 79 years with a histological diagnosis of CRC who attended the North IREN, as well as non-CRC patients from the TRTH surgical outpatient clinic. North IREN is the referral center for cancer patients at hospitals in Northern Peru, including TRTH. Patients with fewer than six teeth, underlying immunosuppression, hereditary CRC (including Lynch syndrome), or those who refused to participate were excluded from the study. Approval from the Faculty of Human Medicine (RESOLUTION No. 4567-2024-FMEHU-UPAO) and the Bioethics Committee of the "Antenor Orrego" Private University (Bioethics Committee Resolution No. 000396-2025-UPAO), the Board of Directors of the Research Ethics Committee of the TRTH (No. 001-2025), and the North IREN Institutional Research Ethics Committee (No. 000225-2025-GRLL-GGR-GRS-IREN) was obtained for this study. These units strictly comply with the principles established in the Declaration of Helsinki, adopted by the 75th General Assembly of the World Medical Association, and the General Health Law of Peru No. 26842. Before requesting their participation, all patients received information about the research purposes. Upon acceptance, they were given the informed consent form to read and sign. The diagnosis of CRC was established or ruled out by specialist physicians from the respective departments through biopsy and appropriate complementary tests. The presence of periodontitis was subsequently evaluated according to the CDC/AAP classification, validated and agreed upon by the Centers for Disease Control and Prevention and the American Academy of Periodontology. This classification requires evaluation of clinical attachment level (CAL) and probing depth (PPD). The North Carolina periodontal probe was used and the following criteria were considered: no periodontitis, mild periodontitis (2 interproximal sites with CAL 3 mm and 2 interproximal sites with PPD 4 mm, not on the same tooth, or 1 interproximal site with PPD 5 mm), moderate periodontitis (2 interproximal sites with CAL 4 mm, not on the same tooth, or 2 interproximal sites with PPD 5 mm, not on the same tooth), and severe periodontitis (2 interproximal sites with CAL 6 mm, not on the same tooth, and 1 interproximal site with PPD 5 mm). Results were recorded on the corresponding data collection form, which also included basic demographic information and information about the other covariates. The reliability of the method for measuring periodontitis using the CDC/AAP index was assessed in the pilot study through intra- and inter-rater calibration (a prior-trained examiner with a specialist professor in Periodontology from the "Antenor Orrego" Private University), with a complete sextant per patient. The same procedure was followed for the dental biofilm and BOP measurement. The intra-rater intraclass correlation for PPD and CAL was estimated at RHO=0.910 and RHO=0.995, respectively; and, for the presence of plaque and BOP, Kappa=0.820 and Kappa=0.938, respectively. Regarding the inter-rater intraclass correlation (examiner-specialist), RHO=0.904 and RHO=0.994 were adopted for PPD and CAL, respectively, and Kappa=0.825 and Kappa=0.934 for plaque presence and BOP, respectively. The collected data were entered into Excel and subsequently processed using IBM SPSS software version 29 (IBM, Armonk, NY, USA). Results are presented in frequency tables, bar graphs, and donut charts. The analysis of the association between the severity of periodontitis and CRC, along with other factors, was conducted using the bivariate Chi-square test, ordinal logistic regression, and binary logistic regression models, with a significance level of p &lt; 0.05.

## Results

**Figure 1:**
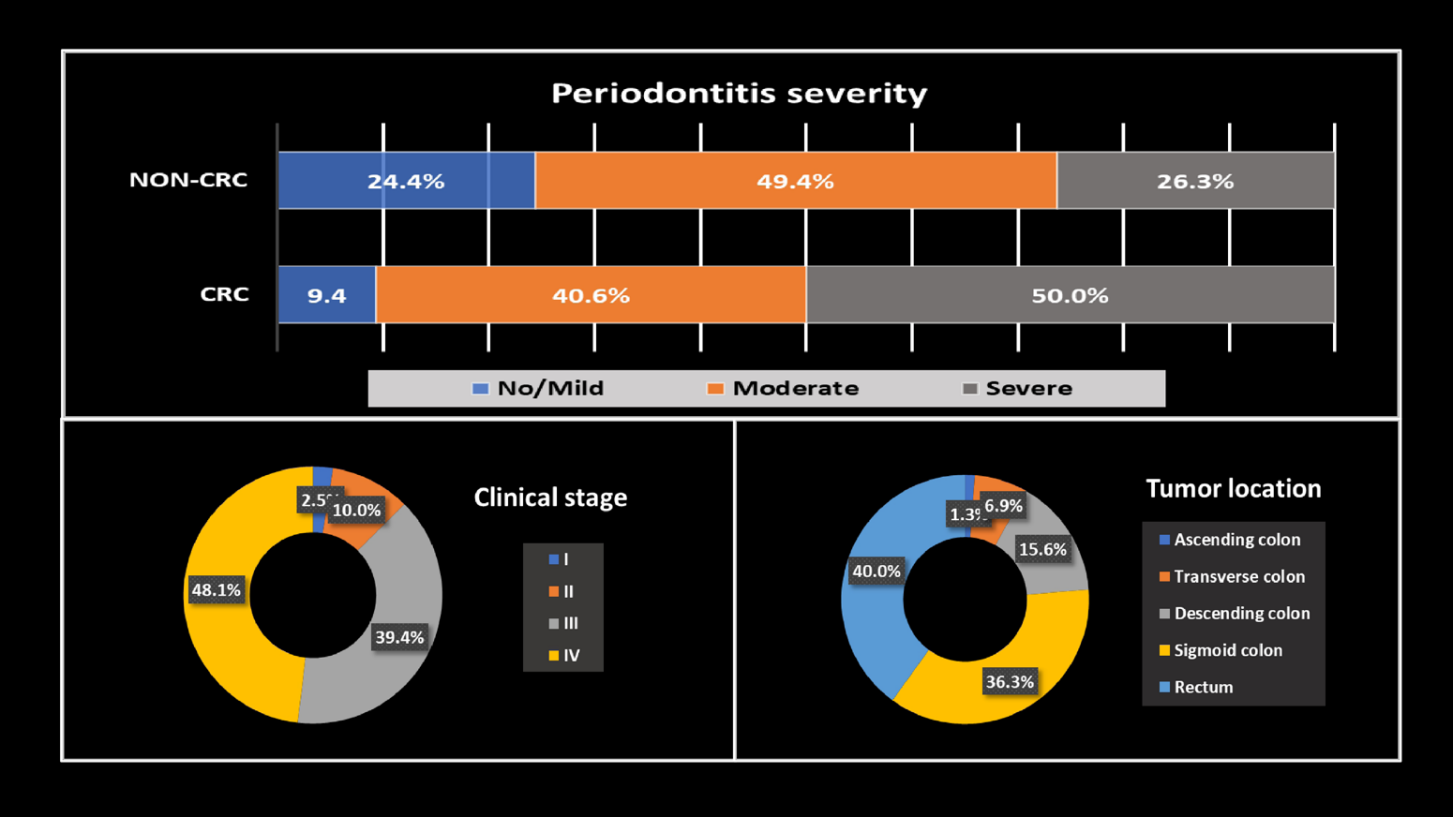
Periodontitis severity by study group, clinical stage and tumor location in CRC patients.

The study included 320 patients, 155 male and 165 female, with mean ages of 58.2 (±11.7) and 55.6 (±11.1) years, respectively. The most common CRC stages were stage IV (48.1%) and stage III (39.4%), and the most common tumor locations were the rectum (40.0%) and sigmoid colon (36.3%), as shown in Figure 1.

1

Furthermore, in non-CRC patients, severe periodontitis was 26.3%, but in CRC patients, it was 50%. Table 1 presents the bivariate analysis of periodontitis severity with CRC and other factors.

Table 1

An association was found between periodontitis severity and CRC (p=0.000), as well as with the other factors studied (p&lt;0.05), except for T2DM (p=0.881). Severe periodontitis was more frequently present in patients aged 60-79 years (54.1%), female gender (59.8%), non-diverticulosis (64.8%) and non-T2DM patients (67.2%), patients with inadequate DPC (85.2%), and BOP &gt;30% (75.4%). Table 2 shows the bivariate analysis, in which CRC was associated with periodontitis severity (p=0.000) and other factors (p&lt;0.05), except gender (p=0.576).

Table 2

In the CRC group, patients with severe periodontitis (50%), age 60-79 years (58.8%), diverticulosis (55%), and daily toothbrushing frequency 1 (73.8%) were predominantly observed. In the non-CRC group, a higher smoking rate was observed (79.4%); while in both groups, lower DPC was observed (85.6% for CRC and 66.9% for non-CRC), lower alcohol consumption (39.4% for CRC and 53.1% for non-CRC), and the majority of non-T2DM patients (61.3% for CRC and 73.8% for non-CRC). Tables 3 and 4 present the multivariate analysis using logistic regression, in which the effect of each factor is evaluated while adjusting for the other covariates, with no association between severe periodontitis and CRC observed in either model.

Table 3

Table 4

Table 3 shows that a higher probability of severe periodontitis in females (p=0.011), in non-smoking patients (p=0.044), with inadequate DPC (p=0.040), in heavy alcohol consumption compared to moderate consumption (p=0.014), as well as in those who do not brush their teeth daily (p=0.045) and in those who brushed twice a day (p=0.049), compared to those who brush three or more times. Greater severity was also observed in those with BOP &gt;30% (p=0.031 for &lt;10% and p=0.000 for 10-30%). While, as observed in Table 4, there was a higher probability of CRC only in patients aged 60-79 years (OR=0.192, p=0.000) and in non-smokers (OR=3.074, p=0.014).

## Discussion

Periodontitis can lead to the release of inflammatory mediators in the blood, such as IL-6 and IL-1, triggering a systemic response. Likewise, opportunistic pathogenic bacteria such as F. nucleatum and P. gingivalis can reach the colon via the bloodstream, producing harmful metabolites that trigger maladaptive immune responses, inflammation, DNA damage, and genomic instability, thereby favoring the onset of pathological processes that could contribute to the development of CRC (1). In the present study, the most frequent CRC stages were IV and III, while in the study by Antonacci et al. (8) (Italy), stages II and III were the most prevalent. This difference may be due to European countries allocating more resources to early cancer prevention and diagnosis (3). Furthermore, these countries are leaders in research against this disease, promoting knowledge exchange and scientific development (9). On the other hand, in Peru, with a weakened healthcare system, cancer treatment is hampered by insufficient infrastructure, diagnostic delays, and inaccessible treatments in public facilities. Moreover, in public attention in Peru, dental care is precarious, with no health policy prioritizing its implementation for cancer patients. In the present study, more than 50% of CRC patients suffered from severe periodontitis at the time of evaluation. These results are consistent with the findings of Antonacci et al. (8) and Idrissi et al. (4), who reported prevalence of 76% and 46%, respectively. It is worth noting that the latter assessed periodontal disease, specifically self-reported gingivitis and periodontitis. The findings of the present study are concerning because cancer treatments such as chemotherapy and radiotherapy generate inflammatory processes in the oral cavity, delaying treatment and increasing medical costs (10). Direct tissue damage and inflammation, along with predisposing factors, can promote the progression of periodontitis if not promptly controlled (11). Likewise, preexisting periodontitis represents a high-risk chronic infectious focus in patients immunosuppressed due to cancer treatment, as it can trigger systemic infections, impair healing, aggravate mucositis, and increase the risk of osteoradionecrosis, especially in the presence of deep periodontal pockets (12). Moreover, limitations in oral hygiene, associated with pain, xerostomia, and tooth loss, negatively affect nutrition, quality of life, and patient recovery. Therefore, periodontal evaluation and treatment before the start of cancer therapy is essential to reduce complications and improve clinical outcomes. Using multivariate analysis, the present investigation found no association between periodontitis severity and CRC, in contrast to the bivariate analysis. It should be noted that multivariate analysis is more potent for simultaneously studying multiple associated factors and their effects. In this sense, bivariate analysis results should always be taken with caution. The results of the present study were consistent with the studies by Nwizu et al. (13), Michaud et al. (14), Kim et al. (15), and Pu et al. (16) However, they differ from the studies by Antonacci et al. (8), Idrissi et al. (4), Hu et al. (17), and Momen-Heravi et al. (18) These discrepancies are possibly due to multiple factors, such as comorbidities, biological characteristics of the tumor and access to health and food systems. The study design and the heterogeneity in the definition of periodontitis could also represent essential limitations. The standard method for diagnosing periodontitis is clinical and/or radiographic, while hospital records, even when specialist dentists perform them, may have certain limitations (2); the same occurs with self-reports, which may be subject to information bias, since patients may forget relevant information or give answers influenced by social norms (1). On the other hand, with respect to covariates, greater periodontitis severity was observed in females, nonsmokers, in those with inadequate DPC, heavy alcohol consumers, in those who do not brush their teeth daily, in those who brush their teeth twice a day, and in those with more than 30% of BOP sites. Women have higher concentrations of salivary inflammatory mediators, increased IL-1 and TNF- levels in the blood (19), and reduced estrogen levels, which negatively affect bone density and periodontal tissues. This hormonal deficiency can weaken dental support and promote inflammation. Heavy alcohol consumption promotes osteoclast formation and periodontal tissue damage (20); furthermore, it impairs neutrophil, macrophage, and T lymphocyte function, increasing susceptibility to infections and the progression of periodontitis (21). BOP, as a clinical indicator of periodontal inflammation, reflects disease activity and is associated with the progression of periodontitis (22). The presence of dental biofilm increases the risk of gingival inflammation and contributes to the loss of periodontal support tissues associated with periodontitis. Furthermore, there is a condition that affects the periodontal apparatus regardless of the presence of dental plaque, caused by neoplastic diseases (23). A strange finding was greater periodontitis severity in nonsmokers; however, these results should be interpreted with caution because these factors are covariates in the present study. Additionally, there was a higher probability of presenting CRC in patients aged 60-79 years and, strangely, also in nonsmokers; however, this last result should be taken with caution due to behavioral changes after the cancer diagnosis or because patients could be changing their responses to avoid criticism. The main limitation of the present study was that it did not allow for causal inference; instead, it only allowed for associations between variables, which should be further assessed in longitudinal studies. Furthermore, although some confounding factors were considered in the evaluation, the authors believe that additional factors may be involved; therefore, we recommend that these be identified and incorporated into future studies using multivariate analyses. On the other hand, because some data, such as alcohol consumption and smoking, were self-reported, they could be subject to the risk of information bias. The examiner's training and calibration are among the strengths of this study, which increase data reliability and ensure greater accuracy of results. Furthermore, the multivariate analysis, along with the large sample size, provides greater statistical power to reach conclusions, accounting for oral and medical factors that are more likely to exert confounding effects. Public health strategies should be established to promote the control of periodontitis in cancer patients, as well as longitudinal studies evaluating the presence of specific periodontopathogenic bacteria as a risk factor for CRC.

## Figures and Tables

**Table 1:** Factors associated with the severity of periodontitis: bivariate analysis.

Factors		Severity of periodontitis						X2	p
		No/mild		Moderate		Severe			
		N°	%	N°	%	N°	%		
Cancer	CRC	15	27.8	65	45.1	80	65.6	23.86	0.000
	Non-CRC	39	72.2	79	54.9	42	34.4		
Age group	40 - 59 years	34	63.0	85	59.0	56	45.9	6.39	0.041
	60 - 79 years	20	37.0	59	41.0	66	54.1		
Gender	Male	25	46.3	81	56.3	49	40.2	6.96	0.031
	Female	29	53.7	63	43.8	73	59.8		
Diverticulosis	No	43	79.6	110	76.4	79	64.8	6.14	0.046
	Yes	11	20.4	34	23.6	43	35.2		
T2DM	No	38	70.4	96	66.7	82	67.2	0.25	0.881
	Yes	16	29.6	48	33.3	40	32.8		
Daily tooth brushing frequency	Does not brush	5	9.3	36	25.0	35	28.7	28.10	0.000
	1 time	10	18.5	47	32.6	42	34.4		
	2 times	30	55.6	56	38.9	41	33.6		
	3 or more	9	16.7	5	3.5	4	3.3		
Cigarette consumption	No	6	11.1	53	36.8	52	42.6	16.93	0.000
	Yes	48	88.9	91	63.2	70	57.4		
Alcohol consumption	No	34	63.0	57	39.6	57	46.7	14.49	0.025
	Mild	14	25.9	50	34.7	35	28.7		
	Moderate	6	11.1	28	19.4	17	13.9		
	Heavy	0	0.0	9	6.3	13	10.7		
DPC	Adequate	17	31.5	25	17.4	18	14.8	7.21	0.027
	Inadequate	37	68.5	119	82.6	104	85.2		
BOP (% sites)	< 10%	7	13.0	2	1.4	3	2.5	66.42	0.000
	10-30%	40	74.1	59	41.0	27	22.1		
	> 30%	7	13.0	83	57.6	92	75.4		

1

**Table 2:** Factors associated with colorectal cancer: bivariate analysis.

Factors		CRC		Non-CRC		X2	p
		N°	%	N°	%		
Periodontitis	No/Mild	15	9.4	39	24.4	23.86	0.000
	Moderate	65	40.6	79	49.4		
	Severe	80	50.0	42	26.3		
Age group	40 - 59 years	66	41.3	109	68.1	23.32	0.000
	60 - 79 years	94	58.8	51	31.9		
Gender	Male	75	46.9	80	50.0	0.31	0.576
	Female	85	53.1	80	50.0		
Diverticulosis	No	72	45.0	160	100.0	118.64	0.000
	Yes	88	55.0	0	0.0		
T2DM	No	98	61.3	118	73.8	5.70	0.017
	Yes	62	38.8	42	26.3		
Daily tooth brushing frequency	Does not brush	58	36.3	18	11.3	53.79	0.000
	1 time	60	37.5	39	24.4		
	2 times	40	25.0	87	54.4		
	3 or more	2	1.3	16	10.0		
Cigarette consumption	No	78	48.8	33	20.6	27.93	0.000
	Yes	82	51.3	127	79.4		
Alcohol consumption	No	63	39.4	85	53.1	12.74	0.005
	Mild	48	30.0	51	31.9		
	Moderate	36	22.5	15	9.4		
	Heavy	13	8.1	9	5.6		
DPC	Adequate	23	14.4	37	23.1	4.02	0.045
	Inadequate	137	85.6	123	76.9		
BOP (% sites)	< 10%	0	0.0	12	7.5	44.60	0.000
	10-30%	41	25.6	85	53.1		
	> 30%	119	74.4	63	39.4		

2

**Table 3:** Factors associated with the severity of periodontitis: multivariate análisis.

Factors		Complete model		
		Effect	Wald	p
Cancer	CRC	0.597	3.338	0.068
	Non-CRC	Reference		
Age group	40 - 59 years	-0.142	0.305	0.581
	60 - 79 years	Reference		
Gender	Male	-0.620	6.396	0.011
	Female		Reference	
Diverticulosis	No	0.448	1.617	0.204
	Yes	Reference		
T2DM	No	-0.497	2.866	0.090
	Yes	Reference		
Daily tooth brushing frequency	Does not brush	1.251	4.008	0.045
	1 time	0.948	2.129	0.145
	2 times	1.235	3.877	0.049
	3 or more	Reference		
Cigarette consumption	No	0.634	4.054	0.044
	Yes	Reference		
Alcohol consumption	No	-0.058	0.012	0.914
	Mild	-0.561	1.094	0.296
	Moderate	-1.508	6.001	0.014
	Heavy	Reference		
DPC	Adequate	-0.636	4.200	0.040
	Inadequate	Reference		
BOP (% sites)	< 10%	-1.602	4.649	0.031
	10-30%	-1.837	30.847	0.000
	> 30%	Reference		

3

**Table 4:** Factors associated with colorectal cancer: bivariate analysis.

Factors		Complete model			
		Effect	Wald	p	OR
Periodontitis	No/Mild	-0.575	0.597	0.440	0.562
	Moderate	-0.706	2.792	0.095	0.494
	Severe	Reference			
Age group	40 - 59 years	-1.649	15.875	0.000	0.192
	60 - 79 years	Reference			
Gender	Male	-0.287	0.497	0.481	0.750
	Female	Reference			
Diverticulosis	No	-35.491	0.000	0.993	0.000
	Yes	Reference			
T2DM	No	0.417	0.563	0.453	1.517
	Yes	Reference			
Daily tooth brushing frequency	Does not brush	21.077	0.000	0.994	1.4E+09
	1 time	20.358	0.000	0.994	6.9E+08
	2 times	18.737	0.000	0.995	1.4E+08
	3 or more	Reference			
Cigarette consumption	No	1.123	6.047	0.014	3.074
	Yes	Reference			
Alcohol consumption	No	1.229	2.943	0.086	3.419
	Mild	-0.016	0.001	0.982	0.984
	Moderate	1.616	2.656	0.103	5.034
	Heavy	Reference			
DPC	Adequate	-0.466	0.793	0.373	0.628
	Inadequate	Reference			
BOP (% sites)	< 10%	-19.970	0.000	0.998	0.000
	10-30%	-0.811	2.330	0.127	0.444
	> 30%	Reference			

4

## Data Availability

The datasets used and/or analyzed during the current study are available from the corresponding author.
